# Communication quality between parents and siblings of children with chronic disorders

**DOI:** 10.1016/j.pecinn.2025.100419

**Published:** 2025-07-08

**Authors:** Caitlin M. Prentice, Anna Aanesen, Amalie Kirkedelen Syverstad, Torun M. Vatne, Krister W. Fjermestad

**Affiliations:** a Department of Psychology, University of Oslo, Norway; b Frambu Competence Centre for Rare Disorders, Siggerud, Norway

**Keywords:** Children, Parents, Chronic disorder, Chronic illness, Siblings, Communication

## Abstract

**Objective:**

This study examines parent-sibling communication during a manual-based group intervention (SIBS) that aims to improve communication quality and well-being for siblings of children with a chronic disorder diagnosis.

**Methods:**

Audio recordings and transcripts of 20 parent-sibling conversations were analyzed using the manual-based tool Verona Coding Definition of Emotional Sequences (VR-CoDES). We measured siblings' negative expressions and parental responses, focusing on how parents applied the SIBS target behaviours of listening, exploring and validating responses.

**Results:**

Siblings raised topics such as heritability of the chronic disorder, differential treatment, and instances of violence and temper tantrums. Siblings' negative expressions were 53 % cues (implicit expressions) and 47 % concerns (explicit expressions). Parents provided space in 74 % of the responses to cues and concerns, meaning they gave space for further disclosure. Within these responses, parents applied the SIBS target behaviours, including exploration (59 %), validation (33 %), and listening (8 %).

**Conclusion:**

Parents mainly provided space and used a warm tone when responding to siblings in the SIBS sessions. Responses included a higher proportion of validation responses and a lower proportion of listening responses compared with previous studies.

**Innovation:**

Parents and siblings of children with chronic disorders face unique challenges that can negatively impact the quality of parent-sibling communication and psychological adjustment in siblings. This study contributes new insight into how parents and siblings communicate in an intervention setting, and how characteristics of the intervention may influence the quality of this communication.

## Highlights

• Siblings of children with chronic disorders have an elevated risk of mental health problems.
• This study looks at how such siblings and their parents communicate during an intervention.
• Parents responded to siblings' emotional expressions with a variety of response types.
• Changes to the intervention were linked to increased parental validation of siblings' concerns.

The experience of growing up with a sibling who has a chronic disorder is relatively common, with prevalence rates estimated between 7 % and 17 % [1]. Childhood chronic disorders represent a wide range of long-lasting or permanent conditions, including both somatic illnesses (e.g., cerebral palsy and congenital heart disease) and neurodevelopmental disorders (e.g., autism and ADHD). Siblings of children with chronic disorders have been found to be at increased risk of poor psychological functioning, including both internalizing problems and externalizing problems [2,3,4].

Such risks may stem from a variety of factors. First, siblings can lack knowledge about and misunderstand the diagnosis, which can provoke anxiety, self-blame, and concern about acquiring the same disorder ([5,6]; [7]). Negative behaviours of the child with the diagnosis, including violence, have also been identified as a source of increased sibling stress [8]. In the case of somatic illnesses such as cancer, siblings can experience significantly raised levels of post-traumatic stress symptoms linked to fear of suffering and death for their brother or sister [9]. Chronic disorders affect the entire family, including changes in relationships, roles, and responsibilities of family members, and can cause disruptions to routines for medical treatments, missing out on outings and vacations, and additional caring responsibilities for siblings and parents [5].

Parents of children with chronic disorders experience higher levels of stress compared to parents of typically developing children due to the physical, financial, and psychosocial demands of caring for a child with a chronic disorder [10]. This heightened stress can have negative psychological outcomes, with parents of children with chronic disorders at a raised risk of mental health problems including depression, anxiety, and post traumatic stress disorder [11,10]. Such problems, as well as increased caregiving responsibilities, can cause parents to be less physically and emotionally available for their typically developing children [8].

## 1 Parent-child communication

Parent-child communication in families of children with chronic disorders is, on average, poorer quality than in other families, with higher levels of hostile, intrusive and withdrawn communication than controls [12]. Siblings have described family communication as challenging, with high levels of conflict [8] and have reported feeling a range of conflicting emotions about their sibling with a disorder [13]. However, they also report avoidance of talking about such issues in order to shield their parents from additional concern and stress [14]. Parents may also inadvertently create a withdrawn communication environment in an effort to shield siblings from difficult or painful topics [15]. This is problematic because the quality of parent-child communication in families of children with chronic disorders is known to affect the well-being and mental health of all family members [16,17].

### 1.1 The SIBS intervention

One way to meet the needs of siblings and parents of children with chronic disorders is to organize support groups where the participants can connect with others in similar situations, share experiences, express feelings, and receive support from peers and clinicians [6,18]. SIBS (short for “siblings”) is a manual-based intervention for siblings aged 8–16 years, with the goal of supporting and enhancing communication between parents and siblings via increased parental listening, exploring, and validating of the siblings' experiences. SIBS group leaders are health professionals or trainee health professionals and undergo two days of training before leading sessions.

**Fig. 1:**
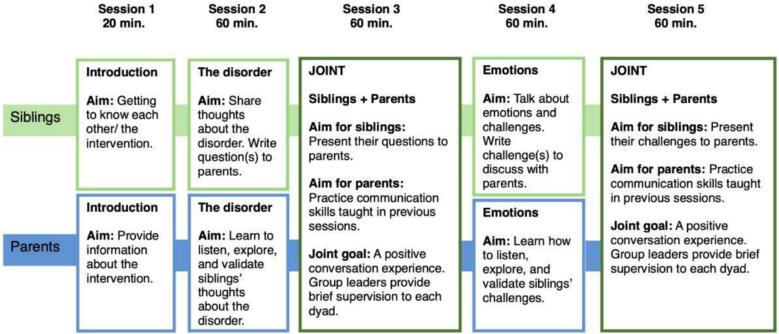
A Randomized Controlled Group Intervention Trial for Siblings of Children with Chronic disorders [19].

The SIBS intervention consists of five sessions (Fig. 1): three group sessions for siblings and parents, conducted separately but with a shared focus, and two joint sessions where siblings and parents engage in 1:1 conversation under supervision [19]. In the sibling sessions, children prepare questions and talking points that they want to discuss in conversation with their parents, with the support of group leaders to identify and articulate the issues they find most important. In the parent groups, the main foci are psychoeducation and communication practice, with the aim of improving parent-child communication. During the joint sessions, one of the group leaders drops in mid-session and gives brief supervision on what the parent can do to improve further.

An open trial study of SIBS found positive effects on communication quality between siblings and parents [16] and a subsequent randomized controlled trial (RCT) found positive, though not statistically significant, effects on sibling mental health and sibling-parent communication [19].

### 1.2 The present study

Between the open trial and RCT, changes were made to training for SIBS group leaders to emphasise the parental response of validating more strongly, as validation was underrepresented in an examination of parent-child conversations during the open trial [20]. This study builds on the open trial Haukeland et al.'s [20] findings by investigating parent-child communication in the SIBS intervention following the changes to group leader training. Our research questions are as follows:

1. How do parents and siblings of children with chronic disorders communicate in an intervention setting?
2. Which topics do children and parents talk about in an intervention setting?
3. To what extent do parents listen, explore, and validate when responding to their sibling child?

Due to the explorative and qualitative nature of the study, we addressed the research questions openly, without a priori hypotheses.

## 2 Methods

### 2.1 Participants

Participants were recruited to join the SIBS-RCT through health care services from three municipalities, five hospitals, a national specialist disorder center for rare disorders, and a range of patient user organizations ([21]). SIBS sessions were in-person. Participant siblings were aged 8–16 years and living with a brother or sister who received specialist and/or municipal health services. The participating siblings did not have a chronic disorder diagnosis themselves.

For the present study, we drew a sub-sample from the SIBS-RCT consisting of 20 siblings and 19 parents (one parent attended separate 1:1 sessions with twin siblings). The dyads included 12 girls and 8 boys and 12 mothers and 7 fathers. Siblings' ages ranged from 8 to 13 years (M = 9). Parents' average education level was high, with 47 % reporting ≥4 years of post-high school education, 37 % reporting 1–4 years of post-high school education, and 16 % completing only high school. A range of chronic disorder diagnoses was represented across participating families, including attention deficit hyperactivity disorder (ADHD), autism spectrum disorder (ASD), Tourette syndrome, eating disorders, cerebral palsy, epilepsy, and intellectual disabilities. Ethnic diversity was low in our sample, as all participants were of European descent.

### 2.2 Design and data collection

Data for our study consists of 20 audio recordings of parent-child dialogues from Session 3 of the SIBS intervention. In Session 3, the focus is on the siblings' questions and thoughts about the disorder, allowing the parents to practice communication skills taught in a previous session. The siblings have prepared questions from Session 2 that they bring into Session 3 and present to their parents, and parents practice responding to these questions by listening, exploring, and validating. At some point during this 1:1 conversation, a group leader enters the room and provides the parent with feedback. Each intervention session had at least two group leaders (one for sibling group and one for parent group) who acted as group leaders, facilitators, and supervisors across the group and 1:1 parts of the intervention. In the complete RCT, there were 28 such group leaders. However, because Session 3 of the intervention was audio recorded rather than video recorded, it is unclear how many individual group leaders were involved in the 1:1 conversations in our sample.

We considered Session 3 appropriate for conducting an in-depth analysis of how parents respond to siblings' cues and concerns because it is the first interaction between the siblings and parents after the parents receive communication training in Sessions 1 and 2.

### 2.3 Analysis

The conversation recordings last around 20 min (m = 24 min) and were transcribed verbatim. From the transcriptions, we noted topics mentioned by siblings, then grouped similar topics into overarching themes. We counted the frequency of each theme, and ranked the themes from most to least frequently mentioned, with the aim of identifying topics of interest to siblings. We continued analyzing new dyads until no new themes were identified and thus the data was considered saturated ([22]).

Parent-sibling communication quality was analyzed using the Verona Coding Definition of Emotional Sequences (VR-CoDES) [23,24], a manual-based coding system designed to study emotional communication between healthcare providers and patients [23]. VR-CoDES was initially designed to identify cues and concerns expressed by adults, but it has also shown efficacy in reliably coding children's emotional expressions and in describing communication quality between parents and children ([[25], [26]]). In the open trial, Haukeland et al. [20] adapted the VR-CoDES for use with SIBS data by dividing “provide space” responses into the additional categories of “listen,” “validate,” and “explore,” which correspond to the target behaviour of the SIBS intervention (Table 1).

**Table 1:** VR-CoDES response category and SIBS target behaviours.

Main category	Subcategory	Code according to SIBS component
Non-explicit provide space	Back-channel	Listening
	Active invitation	Exploring
	Acknowledgement	Validating
	Implicit empathy	Validating
Explicit provide space	Content explore	Exploring
	Affect explore	Exploring
	Content acknowledgement	Validating
	Affect acknowledgement	Validating
	Empathy	Validating
Non-explicit reduce space	Ignore	N/A
	Shutting down	N/A
	Information advice	N/A
Explicit reduce space	Switching	N/A
	Postponement	N/A
	Information advice	N/A
	Active blocking	N/A

The VR-CoDES presents a consensus-based definition of a person's implicit signals or hints (cues) and explicitly expressed emotional distress (concerns) [23,24]. A cue is a verbal or non-verbal hint that suggests an underlying unpleasant emotion and requires clarification. For example, a cue can be statements such as “My brother often gets angry at me” or “Mom and Dad don't spend that much time with me.” A concern is defined as “a clear and unambiguous expression of an unpleasant current or recent emotion where the emotion is explicitly verbalized” ([24], p. 144). Examples of concerns can include statements such as: “I get scared when my brother gets angry at me” or “I feel lonely.” For additional examples of cues and concerns from siblings of children with chronic disorders, see Table 2.

**Table 2:** Examples of negative expressions from siblings and parent responses.

	VR-Codes	Definition	Examples
Negative expression	Cue	The emotion or current is not clearly verbalized or might be present	“I don't bring friends home when my sister is home” “Can I be infected by it?”
	Concern	Clear verbalization of an unpleasant emotional state or current	“It's sad that you always spend time with him and not me” “It annoys me so much”
Response	Provide space	Gives space for further disclosure of the negative expression by the patient. Contextual aspects of the experience or emotion (thoughts, feelings and behaviour) may be explored	“If you would bring your friends home when he is there, how would that be for you?” “Is that something you worry about, getting the illness?”
	Reduce space	Reduces space for further disclosure of the experience	“We spend time with you too, don't you think?” “Don't worry about that!”

VR-CoDES is intended to be applied to responses that occur directly after a cue or concern is expressed [23]. Responses are categorized as providing space or reducing space and as explicit or non-explicit. By providing space, healthcare professionals – or in the case of our study, parents – enable patients – or in the case of our study, their children – to continue sharing and expressing their feelings. When reducing space, parents may respond in a way that reduces space for further disclosure. Non-explicit responses do not explicitly mention either the content or the emotion of the cue or concern, while explicit responses specifically mention either the content or the emotion in the cue or concern.

In this study, coding of parent-child dialogues was carried out by identifying cues, concerns, and repeated cues from participating children, and categorizing parent responses as provide versus reduce space, and as explicit versus non-explicit. Additionally, provide space responses were coded under the subcategories of listening, exploring, or validating responses. Explicit delayed responses from parents and repeated cues were also coded (Table 3). All coding was carried out using NVivo software.

**Table 3:** Overview over coding scheme.

Codes	Subcodes	Subcodes
Cues	N/A	N/A
Concerns	N/A	N/A
Provide space	Explicit	Listening
		Exploring
		Validating
	Non-explicit	Listening
		Exploring
		Validating
Reduce space	Explicit	N/A
	Non-explicit	
Delayed responses	N/A	N/A
Repeated cue	N/A	N/A

Finally, two members of the research team who were also clinicians noted their clinical observations and impressions from the dyads throughout analysis, in response to concerns that the VRCoDES were missing important aspects of parent-child communication in SIBS [20]. Specifically, clinician researchers focused on non-verbal communication, tone of voice, and parents' responses beyond their immediate responses to the siblings' cues and concerns.

## 3 Results

### 3.1 Sibling-selected topics

Siblings raised the following themes in conversation with their parents (listed most to least frequent):

1. Illness: how the sibling got the diagnosis, symptoms and prognosis. Example: *Will he/she get better?* (16 dyads)
2. Differential treatment. Example: *He gets to play more videogames than me*. (9 dyads)
3. Heritability and worries about getting the diagnosis. Example: *I've noticed I have the same things as her. Does that mean I also have the diagnosis?* (7 dyads)
4. Violence, temper tantrums and acting out of the child with a chronic disorder. Example: *She hits me hard when she gets angry*. (7 dyads)
5. Sibling relationship. Example: *I cannot talk with him, he doesn't listen.* (2 dyads)
6. Longing for more time together as a family. Example: *I wish we could go on more holidays together.* (2 dyads)
7. Need for attention, feelings of loneliness. Example: *I never get any attention from dad and mom.* (2 dyads)
8. Impact on social life. Example: *It's embarrassing when she does that in front of my friends.* (2 dyads)

### 3.2 Parent - child communication

#### 3.2.1 Siblings' expression of negative affect

Siblings made a total of 177 expressions of negative affect, with cues accounting for 53.1 % (*n* = 94) and concerns accounting for 46.9 % (*n* = 83). Mean number of cues per dyad was 4.7 (SD = 3.2) and mean number of concerns was 4.15 (SD = 4.8), with considerable variability between dyads. The number of siblings' cues ranged from 1 to 14, while concerns ranged from 1 to 17. Examples of cues included questions like “Is it hereditary?” and “Can it be cured?” as well as statements like “You spend more time with (sibling with a disorder) than with us,” and “I don't want a brother like that.” Examples of concerns included statements such as: “I am afraid that I might get toxic diseases,” “He gets angry and then I get scared,” and “You almost never drive me to practice or come to my games. That is sad.”

#### 3.2.2 Parental responses

A total of 186 parent responses to siblings' cues and concerns were coded. There was a larger proportion of providing space responses (74 %, *n* = 109) compared to reducing space responses (26 %, *n* = 49). However, there was considerable variability between dyads, with providing space responses ranging from 1 to 25 and reducing space responses ranging from 0 to 7.

#### 3.2.3 Provide space responses

Of provide space responses, 80 % were explicit (n = 109). The most common explicit provide space response type was exploring (67 %, *n* = 73). In one example, a sibling asked: “What I'm wondering is if my brother can get better when he gets older, or won't he?” Her mother explicitly explored this content by asking: “Becoming better, what did you have in mind then? Is it something you wish that he was able to do?”

Non-explicit provide space responses accounted for 20 % (*n* = 28) of the total. The most common non-explicit provide response was listening (39 %). Parents could, for instance, non-explicitly listen to cues and concerns by using small prompts or words such as “mmm” and “ok” (referred to as *back-channeling* in the VR-CoDES), to encourage the child to provide more information.

#### 3.2.4 Reduce space responses

Overall, the proportion of reduced space responses was lower than for provide space responses (26 %, *n* = 49). Of the reduce space responses, 77 % were coded as explicit (*n* = 38). A recurring reduce space response style we observed was explicitly responding to the cue or concern by providing information, advice, or reassurance (referred to as *information-advice* in the VR-CoDES manual). For example, in one of the dyads, a sibling asked: “Is he going to be better?” The mother responded: “We don't know that. But it can be better by him and us getting used to living with it as well as handling it, and that we find ways to make it easier for him and us.” Parents were also observed to reduce space by redirecting (referred to as *shifting* in the VR-CoDES manual) the focus away from the cue or concern. This example illustrates how a father reduced space by shifting away from his child's concern:

Parent: “Honestly, why do you think she hits when you get close?”

Sibling: “Because she is happy.”

Parent: “Because she is happy? So, it is actually a good thing?”

Sibling: “No!”

Parent: “Yes, it actually is. I think you didn't quite understand what I meant.”

Sibling: “Yes, but not for me.”

A lower proportion of the reduce space responses were non-explicit (23 %). These types of responses also tended to provide information, advice, and reassurance. For example, a father asked: “What bothers you the most?” The son answered with a concern: “That he hits me.” The father responded: “Mmm, but we are trying to fix that.”

#### 3.2.5 Parental listening, exploring and validating

Provide space responses were further analyzed into listening, exploring and validating, the target behaviours of the SIBS intervention. Exploration, for example asking the child follow-up or clarification questions, was the most frequently used approach (59 %, *n* = 81), with 15 out of 20 dyads using this approach most frequently. Validation was the second most-used SIBS target behaviour (33 %, *n* = 45). We observed parents validate non-explicitly, without referring directly to the cue or concern, such as by saying, “I understand,” as well as validating explicitly, for example by referring directly to the negative emotion that was expressed (e.g. “It is a bit scary when he is angry, I completely understand that.” Listening accounted for 8 % of the provide space responses (*n* = 11), making it the least used SIBS target behaviour. Parents were observed to listen by using small prompts or words after a sibling had expressed a cue or concern. For example, one sibling expressed a concern related to unequal treatment:

We are not allowed to watch TV as much as he is, and we are not allowed to watch TV at the dinner table, and we don't always have someone who has time to put us to bed, but he always has someone who puts him to bed, because he's trying… to get all the attention and spend most of his time with you.

The mother responded with a warm tone in her voice, saying: “Yes” after a pause.

### 3.3 Observed communication quality

Throughout the analyses, we observed a number of instances where the VR-CoDES did not capture important aspects of parent-child communication, including cases of parents practicing the SIBS target behaviours. For example, some parents reduced space, but used a warm tone and asked exploratory questions, which encouraged the sibling to share more. The following conversation between father and daughter illustrates a sibling continuing to share more even though her father used a response that was coded as reduce space according to the VR-CoDES:

Sibling: “But I don't feel like explaining every single time I have visitors.”

Parent: “Do you have visitors that often?”

Sibling: “No, I'm not allowed because [sibling] is home.”

Parent: “What do you think about that?”

Sibling: “That it is very unfair.”

In other cases, the VR-CoDES guidelines prevented instances of parental exploring, validating and listening to be missed in the analysis. For example, following VR-CoDES guidelines, the same cue or concern should not be coded twice, and parental responses should not be coded unless they directly follow an expressed cue or concern. In the conversation below, the parent validates the sibling's thoughts, but this was not captured in the analysis because no cue or concern was presented first:

Sibling: I haven't thought much about it.

Parent: No. There is no right or wrong answer here.

Sibling: Only right answers.

Parent: No, yes. There are only right answers, you could say; that's a better way to put it. Good. Good thinking. Only right answers.

Parents also showed listening behaviour that was not captured by the VR-CoDES, prompting with minimal words such as “yes” and “hmm” while siblings were speaking, instead of showing these behaviours after siblings had finished stating a cue or concern. Finally, sometimes the VR-CoDES led to the recording of a parental response as positive (i.e. one of the target SIBS behaviours) but the tone of the conversation was not positive.

For example, in one of the dyads, a daughter reacted to her father's exploration via questionning:

Parent: “I'm just asking you questions.”

Sibling: “No, it's like an interview.”

## 4 Discussion

The aim of this study was to examine how siblings and parents of children with chronic disorders communicate in an intervention setting. We found that siblings raised a variety of topics with their parents, and that their expressions of negative emotion were split approximately evenly between cues and concerns. Parents mainly responded by providing space; within these responses, exploration was the most common type of SIBS intervention target behaviour, followed by validation and listening.

The topics raised by siblings were similar to those found in previous literature. The most frequently occurring topic was the diagnosis, including worries about contagion or heritability of the disorder. This reflects previous findings that siblings often receive limited information about their sibling's condition and are concerned about their own health in relation to the condition ([5]; [7]). Siblings in our study also expressed dissatisfaction with differential treatment between children in the family, discomfort related to difficult or violent behaviour on the part of their sibling with a diagnosis, and a wish to spend more time together with their parents. These findings build on previous studies showing that having a child with a chronic disorder can result in disproportionate attention and care between different children in the family, stress due to violent behaviour, and the desire for a more “normal” life [27,13,8].

The participating siblings expressed themselves with a more even ratio of cues and concerns (53 % cues and 47 % concerns) than expected from previous applications of VR-CoDES on child data. For example, [25] found that in 16 patient-doctor consultations, children's worries were expressed as 91 % cues and 9 % concerns. However, this study focused on children with cancer communicating with healthcare personnel. It is reasonable to expect that siblings express negative emotions in a more direct manner (i.e. as concerns) when communicating with their parents, due to feeling more comfortable with them than with healthcare personnel. [28] found results more similar to those in our study (59 % cues and 41 % concerns) when applying the VR-CoDES to a different sample of siblings and SIBS group leaders. This higher rate of concerns may have been because group leaders are trained in communication techniques to encourage siblings to express their emotions and experiences. Interestingly, however, Haukeland et al. [20], who also examined parent-child communication in a different sample from the SIBS intervention, found that concerns accounted for only 29 % of expressed emotions. As discussed below, we also found differences in how parents applied the SIBS target behaviours compared to Haukeland et al. [20], with a higher ratio of validating responses in the present study. It is possible that this increased validation may have led to siblings in the current sample feeling more acknowledged and understood, encouraging them to express concerns more openly.

Within their responses to siblings' cues and concerns, we found that parents mainly provided space (74 % of responses), encouraging them to share more about their experiences, situations, and feelings. These results are consistent with findings from Haukeland et al. [20], who also found that parents mainly provided space (69 % of responses). This can be regarded as positive, since facilitating space for children to express themselves is an essential goal of the SIBS training and supportive of open family communication and better sibling mental health. Within provide space responses, we found that exploration was the most frequent of the SIBS target behaviours, which is also in line with results from Haukeland et al. [20]. This may reflect parents' attempts to clarify siblings' cues and potential underlying negative emotions. However, it may also reflect instances where parents appeared to struggle to maintain the conversation with siblings who showed a lack of interest and/or were not accustomed to discussing sensitive issues with their parents [20].

Validating responses accounted for a third (33 %) of the provide space responses in our analyses, making validation the second most used SIBS target behaviour. This finding differs notably from the Haukeland et al. [20] study, where the rate of validation responses was only 8 %. They argued that this may have been due to too little attention being given to validation in the SIBS parent sessions, sparking an increased emphasis on validation in SIBS group leader training and providing the impetus for our follow-up study. Our findings suggest that this increased emphasis in training may have resulted in more use of validating responses by parents during the intervention, although further study is needed before drawing definitive conclusions.

Listening responses accounted for only 8 % of the provide space responses in our analysis, making it the least-used approach. This low rate of listening responses is also inconsistent with Haukeland et al.'s [20] findings, where listening was the second most used response style (16 %). This variation might be explained by different interpretations of the coding manual. In our reading of the VR-CoDES, listening should only be coded if the parent encouraged the sibling to say more using minimal prompts or words, but not full statements. Moreover, for a response to be coded as listening, it must appear after a pause from the expressed cue or concern and not throughout or on top of the child's speech. We noticed that parents in our sample often demonstrated active listening *while* the sibling spoke, making small noises of agreement (e.g. “hmm”) over the top of sibling speech. Following the VR-CoDES, these responses were not coded as listening, despite reflecting the parent showing attentiveness to their child. Consequently, the differences between listening rates found in our study and Haukeland et al. [20] should be interpreted with caution, due to our strict application of the VR-CoDES protocol.

### 4.1 Strengths, limitations and implications

The current study used quantitative analysis combined with qualitative observations of parent-sibling conversations to examine how siblings express themselves in an intervention setting and how parents respond to these expressions. The intervention setting means that our findings do not necessarily reflect how parents and siblings communicate on a day-to-day basis, as parents had received psychoeducation, practiced communication techniques, and accessed the prepared questions from the siblings in advance of the sessions we analyzed.

However, our findings do offer insight into how parents and siblings communicate during the SIBS intervention—an important step towards creating an effective, evidence-based intervention for siblings of children with chronic disorders. One of our most important findings was that parental validation responses increased since a previous study of the SIBS intervention [20], suggesting that changes to group leader training made a difference to intervention outcomes. The mechanisms of this change are unclear and could make a useful area of future study. This study also uncovered topics raised by siblings during the parent-sibling conversations, which can be used to inform development of parent psychoeducation sessions.

The use of VR-CoDES was a key strength of our study, providing a manual-based, quantitative measure of emotional expression and response which allowed for a relatively objective assessment of parent-sibling communication quality and made comparison with results from previous studies possible. However, the VR-CoDES also failed to capture some important aspects of parent-child communication, including active listening behaviours that occurred throughout a child's speech, or parental input that did not follow a cue or concern. Moreover, the VR-CoDES do not cover non-verbal responses and our use of audio recordings did not allow for this to be noted. Parents may have expressed non-verbal validation, attentiveness, or other empathic signals such as supportive facial expressions, hugs, or nodding of their heads that was not captured in our analysis ([29]; [20]). Future studies could use video recordings of parent-child conversations to incorporate the analysis of non-verbal communication. This would place a higher demand on technical aspects of data collection such as video quality and camera angle, which can be challenging in clinical or intervention settings, but would provide more nuanced insight into parental responses.

An additional limitation is that participant parents were, on average, better educated than the general population. Parents with higher levels of education are known to make less use of negative disciplinary practices, have greater knowledge about child development, and engage in more conversations with their children compared to parents with lower levels of education ([[30], [31]]). Consequently, our findings may not be generalizable to families where parents have lower levels of education.

Similarly, our sample included families with a child with a range of chronic disorders including ASD, ADHD, Tourette syndrome, eating disorders, cerebral palsy, epilepsy, and intellectual disabilities, but it is unclear whether our findings would generalize to families with other diagnoses. The presence of somatic illnesses such as cancer, for example, could potentially cause siblings to bring up different themes in conversation, which could in turn provoke different types of responses from parents. Larger study samples representing a wider range of diagnoses could begin to address these questions. Future studies could also explore ways in which the SIBS communication skills could be provided to families in different ways, such as via an online format (see, for example, [32]).

## 5 Conclusion

The current study contributes valuable qualitative insight to a sibling intervention setting. Most parent-sibling dyads communicated with warmth and care, even when using reducing space responses, which facilitated a space for siblings to share their emotions and experiences even if these aspects of communication were not always captured by the VR-CoDES. These results are promising, as one of the key aims of the SIBS intervention is to improve family communication quality by enhancing parental listening, exploration, and validation. Overall, the findings from this study contribute to the further development of the SIBS intervention and to a broader understanding of parent-child communication in families with a child with a chronic disorder.

## CRediT authorship contribution statement

**Caitlin M. Prentice:** Methodology, Supervision, Writing – review & editing, Conceptualization. **Anna Aanesen:** Writing – review & editing, Conceptualization, Formal analysis, Writing – original draft. **Amalie Kirkedelen Syverstad:** Writing – original draft, Writing – review & editing, Conceptualization, Formal analysis. **Torun M. Vatne:** Funding acquisition, Conceptualization. **Krister W. Fjermestad:** Supervision, Writing – review & editing, Conceptualization, Funding acquisition.

## Ethics

The SIBS-RCT was approved by The Regional Committee for Medical and Health Research Ethics South East (REK - 2018/2461) ([21]). Participation in the study was voluntary, and participants gave informed consent to participate.

## Funding

The Norwegian Research Council, project no. 321027.

## Declaration of competing interest

We have no known conflict of interest to disclose.

## Figures and Tables

**Table:** 

Dyad	Cues	Concerns	Provide space	Listening	Explore	Validate	Reduce space
*Dyad 1*	6	14	85 % (17)	10 % (2)	20 % (4)	55 % (11)	15 % (3)
*Dyad 2*	2	3	100 % (5)	0 % (0)	40 % (2)	60 % (3)	0 % (0)
*Dyad 3*	3	7	50 % (5)	0 % (0)	40 % (2)	60 % (3)	50 % (5)
*Dyad 4*	8	5	53 % (7)	14 % (1)	72 % (5)	14 % (1)	46 % (6)
*Dyad 5*	6	9	100 % (14)	0 % (0)	57 % (8)	43 % (6)	0 % (0)
*Dyad 6*	2	1	100 % (3)	0 % (0)	100 % (3)	0 % (0)	0 % (0)
*Dyad 7*	4	2	67 % (4)	0 % (0)	75 % (3)	25 % (1)	33 % (2)
*Dyad 8*	7	1	50 % (8)	25 % (1)	50 % (2)	25 % (1)	50 % (4)
*Dyad 9*	14	17	83 % (25)	4 % (1)	80 % (20)	16 % (4)	17 % (5)
*Dyad 10*	2	1	100 % (3)	33 % (1)	33 % (1)	33 % (1)	0 % (0)
*Dyad 11*	1	3	100 % (7)	14 % (1)	57 % (4)	29 % (2)	0 % (0)
*Dyad 12*	5	1	100 % (6)	33 % (2)	50 % (3)	17 % (1)	0 % (0)
*Dyad 13*	6	6	57 % (8)	0 % (0)	63 % (5)	38 % (3)	42 % (6)
*Dyad 14*	8	0	62 % (5)	0 % (0)	60 % (3)	40 % (2)	37 % (3)
*Dyad 15*	1	1	50 % (1)	0 % (0)	100 % (1)	0 %	50 % (1)
*Dyad 16*	1	0	100 % (2)	0 % (0)	50 % (1)	50 % (1)	0 % (0)
*Dyad 17*	4	8	64 % (9)	11 % (1)	67 % (6)	22 % (2)	35 % (5)
*Dyad 18*	7	2	41 % (5)	20 % (1)	80 % (4)	0 % (0)	58 % (7)
*Dyad 19*	4	2	83 % (5)	0 % (0)	60 % (3)	40 % (2)	16 (1)
*Dyad 20*	3	0	66 % (2)	0 % (0)	100 % (2)	0 %	33.% (1)
*Total*	94	83	74 % (109)	8 % (11)	59 % (81)	33 % (45)	26 % (49)

## References

[1] McKenzie Smith M., Pinto Pereira S., Chan L., Rose C., Shafran R. (2018). Impact of well-being interventions for siblings of children and young people with a chronic physical or mental health condition: a systematic review and meta-analysis. Clin. Child. Fam. Psychol. Rev..

[2] Marquis S.M., McGrail K., Hayes M.V. (2019). A population-level study of the mental health of siblings of children who have a developmental disability. SSM - Popul. Health.

[3] Martinez B., Pechlivanoglou P., Meng D., Traubici B., Mahood Q., Korczak D. (2022). Clinical health outcomes of siblings of children with chronic conditions: a systematic review and Meta-analysis. J. Pediatr..

[4] Pinquart M. (2023). Behavior problems, self-esteem, and prosocial behaviour in siblings of children with chronic physical health conditions: an updated meta-analysis. J. Pediatr. Psychol..

[5] Havill N., Fleming L.K., Knafl K. (2019). Well siblings of children with chronic illness: a synthesis research study. Res. Nurs. Health.

[6] Lobato D.J., Kao B.T. (2002). Integrated sibling-parent group intervention to improve sibling knowledge and adjustment to chronic illness and disability. J. Pediatr. Psychol..

[7] Vatne T.M., Helmen I.Ø., Bahr D., Kanavin Ø., Nyhus L. (2015). She came out of mum’s tummy the wrong way (Mis)conceptions among siblings of children with rare disorders. J. Genet. Couns..

[8] Schumann A., Vatne T.M., Fjermestad K.W. (2024). What challenges do siblings of children with chronic disorders express to their parents? A thematic analysis of 73 sibling-parent dialogues. J. Pediatr. Nurs..

[9] Alderfer M.A., Labay L.E., Kazak A.E. (2003). Brief report: does posttraumatic stress apply to siblings of childhood Cancer survivors?. J. Pediatr. Psychol..

[10] Pinquart M. (2018). M. Stress and health. J. Intern. Soc. Investig. Stress.

[11] Carmassi C., Corsi M., Bertelloni C.A., Pedrinelli V., Massimetti G., Peroni D.G. (2019). Post-traumatic stress and major depressive disorders in parent caregivers of children with a chronic disorder. Psychiatry Res..

[12] Murphy L.K., Murray C.B., Compas B.E., Cynthia A., Gerhardt C.A.B., Wiebe Deborah J. (2017). Topical review: integrating findings on direct observation of family communication in studies comparing pediatric chronic illness and typically developing samples. J. Pediatr. Psychol..

[13] Haukeland Y.B., Fjermestad K.W., Mossige S., Vatne T.M. (2015). Emotional experiences among siblings of children with rare disorders. J. Pediatr. Psychol..

[14] Nygård C., Clancy A., Kitzmüller G. (2024). Balancing on life’s ladder: a meta-ethnography of the existential experiences of siblings of children with complex care needs. J. Adv. Nurs..

[15] Deavin A., Greasley P., Dixon C. (2018). Children’s perspectives on living with a sibling with a chronic illness. Pediatrics.

[16] Haukeland Y.B., Czajkowski N.O., Fjermestad K.W., Silverman W.K., Mossige S., Vatne T.M. (2020). Evaluation of “SIBS”, an intervention for siblings and parents of children with chronic disorders. J. Child Fam. Stud..

[17] Zapf H., Boettcher J., Haukeland Y., Orm S., Coslar S., Wiegand-Grefe S. (2023). A systematic review of parent–child communication measures: instruments and their psychometric properties. Clin. Child. Fam. Psychol. Rev..

[18] Wolff B., Magiati I., Roberts R., Skoss R., Glasson E.J. (2023). Psychosocial interventions and support groups for siblings of individuals with neurodevelopmental conditions: a mixed methods systematic review of sibling self-reported mental health and wellbeing outcomes. Clin. Child. Fam. Psychol. Rev..

[19] Kirchhofer S.M., Fredriksen T., Orm S., Botta M., Zahl E., Cogo-Moreira H. (2025). Effectiveness of a group intervention to improve mental health in siblings of children with chronic disorders: a cluster randomized controlled trial. J. Pediatr. Psychol..

[20] Haukeland Y.B., Fjermestad K.W., Mossige S., Vatne T.M. (2022). Parent-child communication about emotions during SIBS: a joint intervention for siblings and parents of children with chronic disorders. Nord. Psychol..

[21] Fjermestad K.W., Silverman W.K., Vatne T.M. (2020). Group intervention for siblings and parents of children with chronic disorders (SIBS-RCT): study protocol for a randomized controlled trial. Trials.

[22] Saunders B., Sim J., Kingstone T., Baker S., Waterfield J., Bartlam B., Burroughs H., &Jinks C. (2018). Saturation in qualitative research: exploring its conceptualization and operationalization. Quality & Quantity.

[23] Del Piccolo L., de Haes H., Heaven C., Jansen J., Verheul W., Bensing J. (2011). Development of the Verona coding definitions of emotional sequences to code health providers’ responses (VR-CoDES-P) to patient cues and concerns. Patient Educ. Couns..

[24] Zimmermann C., Del Piccolo L., Bensing J., Bergvik S., De Haes H., Eide H. (2011). Coding patient emotional cues and concerns in medical consultations: the Verona coding definitions of emotional sequences (VR-CoDES). Patient Educ. Couns..

[25] Vatne T.M., Finset A., Ørnes K., Ruland C.M. (2010). Application of the Verona coding definitions of emotional sequences (VR-CoDES) on a pediatric data set. Patient Educ. Couns..

[26] Vatne T.M., Ruland C.M., Ørnes K., Finset A. (2012). Children’s expressions of negative emotions and adults’ responses during routine cardiac consultations. J. Pediatr. Psychol..

[27] Chan G.W.L., Goh E.C.L. (2014). ‘My parents told us that they will always treat my brother differently because he is autistic’ – are siblings of autistic children the forgotten ones?. J. Soc. Work. Pract..

[28] Vatne T.M., Zahl E. (2017). Emotional communication in support groups for siblings of children with disabilities. Patient Educ. Couns..

[29] Fruzzetti A.E., Shenk C. (2008). Fostering Validating Responses in Families. Social Work in Mental Health.

[30] Rowe M.L. (2008). Child-directed speech: relation to socioeconomic status, knowledge of child development and child vocabulary skill. J. Child Lang..

[31] Bøe T., Sivertsen B., Heiervang E., Goodman R., Lundervold A.J., Hysing M. (2014). Socioeconomic Status and Child Mental Health: The Role of Parental Emotional Well-Being and Parenting Practices. J. Abnorm. Child Psychol..

[32] Zeleznik T., Thompson R., Vatne T., Fjermestad K., Limbers C. (2025). SIBS-ONLINE: satisfaction of an online program for siblings and parents of children living with type 1 diabetes. Diabetes Technol. Obes. Med..

